# Role of Elevated Intracellular S-Adenosylhomocysteine in the Pathogenesis of Alcohol-Related Liver Disease

**DOI:** 10.3390/cells9061526

**Published:** 2020-06-23

**Authors:** Madan Kumar Arumugam, Sharanappa Talawar, Laura Listenberger, Terrence M. Donohue, Natalia A. Osna, Kusum K. Kharbanda

**Affiliations:** 1 Research Service, Veterans Affairs Nebraska-Western Iowa Health Care System, Omaha, NE 68105, USA; madankumar.arumugam@unmc.edu (M.K.A.); sharanappa.talawar@unmc.edu (S.T.); tdonohue@unmc.edu (T.M.D.J.); nosna@unmc.edu (N.A.O.); 2 Department of Internal Medicine, University of Nebraska Medical Center, Omaha, NE 68198, USA; 3 Departments of Biology and Chemistry, St. Olaf College, Northfield, MN 55057, USA; listenbe@stolaf.edu; 4 Department of Biochemistry & Molecular Biology, University of Nebraska Medical Center, Omaha, NE 68198, USA

**Keywords:** hepatic steatosis, S-adenosylhomocysteine, alcohol, lipid droplets, lipases, perilipins, hepatocyte, 3-deazaadenosine

## Abstract

Background: The earliest manifestation of alcohol-related liver disease (ALD) is steatosis, characterized by the accumulation of lipid droplets (LDs) in hepatocytes. Findings from our laboratory have indicated that many pathological changes, including steatosis, correlate with the alcohol-induced hepatocellular increases in S-adenosylhomocysteine (SAH). Based on these considerations, we hypothesized that an experimental increase in intracellular SAH alone will result in similar steatotic changes to those seen after alcohol exposure. Methods: Freshly isolated rat hepatocytes grown on collagen-coated plates were exposed to serum-free medium containing 50 µmol/L oleic acid and varying concentrations of 3-deazaadenosine (DZA) to experimentally elevate intracellular SAH levels. Results: Overnight exposure to DZA treatment dose-dependently increased hepatocellular triglyceride accumulation, which was also evident by morphological visualization of larger-sized LDs. The rise in triglycerides and LDs accompanied increases in mRNA and protein levels of several LD-associated proteins known to regulate LD number and size. Furthermore, DZA treatment caused a decline in the levels of lipases that prevent fat accumulation as well as increased the expression of factors involved in lipogenesis and fatty acid mobilization. Collectively, our results indicate that the elevation of intracellular SAH is sufficient to promote fat accumulation in hepatocytes, which is similar to that seen after alcohol exposure.

## 1. Introduction

The earliest manifestation of alcohol-induced liver injury is steatosis, characterized by fat accumulation in this organ, which, histologically, is seen as higher numbers and larger sizes of specialized organelles called lipid droplets (LDs) in the cytoplasm of the hepatocyte [1,2]. LDs are the main storage depots for intracellular lipids [3]. Although once considered inert, LDs are metabolically active, highly dynamic organelles, critically important for maintaining lipid homeostasis and preventing fat accumulation in the liver [4,5,6]. Structurally, LDs are composed of a core of neutral lipid including triglycerides (TGs) and cholesteryl esters. The neutral lipid core is shielded from the surrounding cytosol by a complex array of surface phospholipids and proteins [7]. The predominant phospholipid that comprises the LD monolayer envelope is phosphatidylcholine (PC) followed by phosphatidylethanolamine (PE) that stabilize and facilitate interactions of LDs with other cellular compartments [8]. The LD surface is decorated with specific proteins, including the perilipin (PLIN) family of proteins (PLIN1 to PLIN5), structural proteins, lipid metabolizing enzymes, membrane trafficking proteins and signaling proteins. This orbit of LD-associated proteins serves important functions in lipid metabolism by regulating lipolysis of the LD TG stores [9,10,11].

Recently, we discovered that many hallmark features of alcoholic liver injury, including accumulation of larger-sized LDs, correlated with a reduced ratio of S-adenosylmethionine (SAM) to S-adenosylhomocysteine (SAH) associated with higher intracellular SAH levels [1,2,12,13,14]. We further reported that the rise in LD size correlated inversely with its surface PC to PE content and that a reduction in the PC:PE ratio caused increased recruitment of PLINs [1], which are negative regulators of LD TG hydrolysis [15,16]. More importantly, strategies designed to prevent SAH elevation, such as betaine administration to ethanol-fed animals, also prevent alcohol-induced LD accumulation [1,2,12,13,14]. Based on these findings, we hypothesized that increases in intracellular SAH alone would result in lipid accumulation similar to that seen after alcohol exposure. To test this, we exposed cultured primary hepatocytes to 3-deazaadenosine (DZA). Such exposure causes intracellular SAH accumulation by blocking the activity of S-adenosylhomocysteine hydrolase [17]. We then determined whether increased intracellular SAH altered LD morphology, number and characteristics.

## 2. Materials and Methods

### 2.1. Animals

All animals received humane care in accordance with the guidelines established by the American Association for the Accreditation of Laboratory Animal Care. All experimental protocols were approved by the Institutional Animal Care and Use Committee at the Nebraska-Western Iowa Health Care System Veterans Affairs Medical Center and complied with NIH guidelines. Male Wistar rats weighing 175–200 g, purchased from Charles River (Raleigh, NC, USA) were maintained on a regular light (12 h)/dark (12 h) cycle and fed a standard laboratory chow diet.

### 2.2. Isolation of Hepatocytes and Experimental Design

Hepatocytes were prepared from the livers of the rats by a modified collagenase-perfusion technique [18,19]. We assessed viabilities of the different cell populations by trypan blue exclusion. Only cell preparations that had viabilities of >90% were used. We plated hepatocytes (0.8 million viable cells/well) onto collagen IV-coated 6 well-plates in Williams’ E medium, supplemented with antibiotics and 5% fetal calf serum, as previously described [18,19]. Two hours after plating and incubation at 37 °C in a humidified atmosphere of 95% O_2_/5% CO_2_, we removed the medium and replaced it with 1.7% BSA-supplemented Williams’ E medium containing 50 µmol/L oleic acid (OA) and zero, 20 µM or 50 µM DZA (Cat#8296, Sigma-Aldrich, St. Louis, MO, USA) for overnight treatment. In the initial stages of this study, we conducted a pilot study to confirm the rise in hepatocellular SAH levels by DZA treatment. We observed a ~1.5- and 3-fold rise in SAH levels over controls, in hepatocytes after overnight exposure to 20 µM or 50 µM DZA, respectively. Our results are in line with those reported by other laboratories [17,20,21].

### 2.3. Estimation of TGs

Total lipids were extracted from the hepatocytes as detailed in our publications [1,12]. TG mass was quantified using the diagnostics kit (Cat#TR22421, Thermo DMA kit, Thermo Electron Clinical Chemistry, Louisville, CO, USA) as detailed [1,12].

### 2.4. Messenger RNA Quantification

Total RNA was isolated from control and DZA- treated cells using a PureLink^TM^ RNA Mini Kit (Cat#12183018A, Invitrogen, Waltham, MA, USA), according to the manufacturer’s instructions. We determined the RNA concentration at A260 and its purity as the A260/280 ratio, using a Nanodrop spectrophotometer (NanoDrop Technologies, Wilmington, DE, USA). Further, a 2-step procedure was applied, in which 200 ng RNA was reverse-transcribed to cDNA using the high capacity reverse transcription kit (Cat#4368813, Applied Biosystems, Waltham, MA, USA). We then amplified the cDNA using TaqMan Universal Master Mix-II (Cat#4440038, Applied Biosystems, Waltham, MA, USA). with fluorescent-labeled FAM primers (TaqMan gene expression systems, Cat#4331182, Applied Biosystems, Waltham, MA, USA). After incubation in a Model 7500 qRT-PCR thermal cycler (Applied Biosystems, Waltham, MA, USA), the relative quantity of each RNA transcript was calculated by its threshold cycle (Ct) after subtracting that of the reference cDNA (β-actin). Data are expressed as the relative (RQ) quantity of each transcript

### 2.5. Western Blotting

Immunoblotting was performed by loading equal amounts of protein from cell lysates onto SDS-PAGE gels, as described in our publications [1,12]. After incubation of the membranes with the appropriate secondary antibodies, proteins were visualized using standard enhanced chemiluminescence detection methods and imaged using a BIORAD ChemiDoc MP imaging system software (Bio-Rad Laboratories, Hercules, CA, USA). The intensities of immunoreactive protein bands were quantified using Quantity One software (Bio-Rad Laboratories, Hercules, CA, USA).

### 2.6. Fluorescence Microscopy

Hepatocytes grown on collagen-coated coverslips under similar culture and overnight treatment conditions as detailed above were processed as follows:

BODIPY Staining of LDs: Coverslips were fixed in 4% *w/v* paraformaldehyde in 50 mM PIPES, pH 7.0, and the accumulated neutral lipids were visualized by staining with 1 µg/mL BODIPY493/503 (Cat#D3922, Invitrogen, Waltham, MA, USA). The cell nuclei were stained with DAPI (1 µg/mL). Cell images were captured using a Keyence BZ-X810 florescence microscope (Plano, TX, USA) and analyzed for quantitating LD numbers and sizes using Keyence BZ-X810 Analyzer software (Plano, TX, USA).

Immunofluorescence Staining: Coverslips were rinsed with PBS and fixed with 4% paraformaldehyde in PBS followed by background quenching with 50 mM NH_4_Cl in PBS. Cells were permeabilized with 0.1% Triton X-100 in PBS for 5 min at RT and blocked with 2% BSA in PBS for 1h at RT. The coverslips were then placed into a humidified chamber and incubated for 2 h at 37 °C with 1 µg/mL each of anti-PLIN2 (Cat#10R-A117AX, Fitzgerald, Acton, MA, USA), anti-fatty acid synthase (Cat#ab22759, Abcam, Cambridge, MA, USA) or anti-sterol regulatory element-binding protein 1 (Cat#ab28481, Abcam, Cambridge, MA, USA) in PBS containing 2% BSA and 0.1% Triton X-100. The coverslips were then washed with PBS and then incubated for 1 h at room temperature with the corresponding Alexa Fluor 647 (Cat#A21235, Invitrogen, Waltham, MA, USA) or Alexa Fluor 488 (Cat#A21206, Invitrogen, Waltham, MA, USA) diluted 1:1000 in PBS containing 2% BSA and 0.1% Triton X-100. After incubation, the coverslips were washed with PBS, stained with DAPI (1 µg/mL) and mounted. We visualized the cells under a Keyence BZ-X810 florescence microscope and captured images. We quantified staining intensity with Keyence BZ-X810 Analyzer software.

Time-Bound Studies: To evaluate the changes in LDs with increasing times of exposure to DZA, we modified the basic culture procedure detailed above as follows. Briefly, freshly isolated hepatocytes plated on collagen-coated coverslips and allowed to attach for 2 h were washed with serum-free Williams’ E medium followed by overnight incubation with 50 µmol/L OA in 1.7% BSA- containing media supplemented with 1 µg/mL BODIPY 558/568 C12 (Cat#D3835, Invitrogen Waltham, MA, USA) for labelling newly-formed LDs. The cells were then thoroughly washed with PBS and incubated for varying times with OA/BSA media containing 1 µg/mL BODIPY FL C12 (Cat#D3822, Invitrogen Waltham, MA, USA) and different concentrations of DZA. After 0.5, 1, 2 and 4 h following the addition of the DZA, the coverslips were washed, fixed with 4% paraformaldehyde, stained with DAPI, mounted and images captured using a Keyence BZ-X810 florescence microscope. Quantification of fluorescence intensity was performed using Keyence BZ-X810 Analyzer software.

### 2.7. Statistical Analyses

All experimental data are expressed as mean values ± standard deviation. We used one-way ANOVA and a Tukey post-hoc test to compare multiple groups. We used Student’s *t*-test for comparisons between two groups. A probability *p*-value of 0.05 or less is considered significant.

## 3. Results

### 3.1. DZA Treatment Causes TG Level Increase and Accumulation of Larger-Sized LDs in Hepatocytes

**Figure 1:**
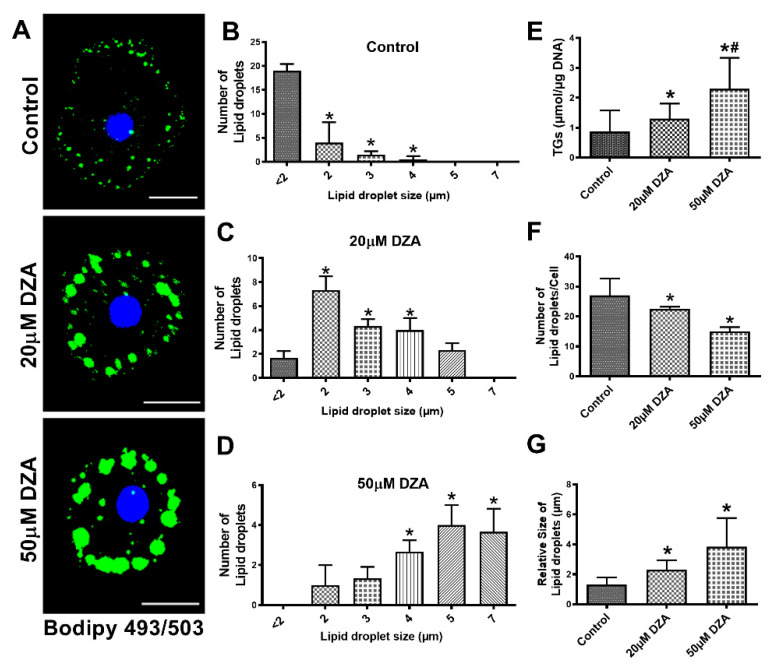
Characterization of LDs in control and DZA-treated hepatocytes. (**A**) Accumulation of LDs in control and DZA-treated hepatocytes determined by BODIPY staining (Scale bar 20 µm). (**B**) Distribution of different sized LDs in control, (**C**) 20 µM DZA, and (**D**) 50 µM DZA-treated hepatocytes, *n* = 4 experiments; images of 30 random cells from each treatment group per experiment were captured and analyzed for their LD numbers and size (in diameter) (* *p* ≤ 0.05 vs. <2 µm). (**E**) Triglyceride levels in control and DZA-treated hepatocytes, *n* = 11 experiments. (**F**) Total number of LDs per cell in control and DZA-treated hepatocytes, *n* = 4 experiments; images of 30 random cells from each treatment group per experiment were captured and analyzed for LD number/cell. (**G**) LD size (in diameter) in control and DZA-treated cells, *n* = 4 experiments; images of 30 random cells from each treatment group per experiment were captured and analyzed for LD size/cell. Asterisks indicate values for DZA-treated hepatocytes are statistically different from controls at * *p* ≤ 0.05 vs. Control; ^#^ *p* < 0.001 vs. Control.

We observed a dose-dependent increase in LD accumulation in hepatocytes treated with DZA, as visualized by BODIPY 493/503 staining (Figure 1A). Quantification of LDs revealed heterogeneity in their sizes and numbers in the hepatocytes. An increasing number of larger-sized LDs were seen in DZA-treated groups compared with unexposed control hepatocytes, in which the majority of LDs were smaller (<2 μm), whereas larger-sized LDs (5–7 μm) comprised the majority of LDs in cells exposed to 50 μM DZA (Figure 1B–D). Biochemical determination of the cellular TG content supported the morphological changes in LDs and revealed a dose-dependent rise with DZA treatments (Figure 1E). While the total number of LDs per hepatocyte decreased (Figure 1F), the average LD size nearly doubled with each increment of DZA treatment (control—1.3 ± 0.13 µm; 20 µM DZA—2.3 ± 0.17 µm; 50 µM DZA—3.8 ± 0.52 µm; *p* ≤ 0.05; Figure 1G).

### 3.2. DZA Treatment Increase PLIN2, PLIN3 and PLIN5

**Figure 2:**
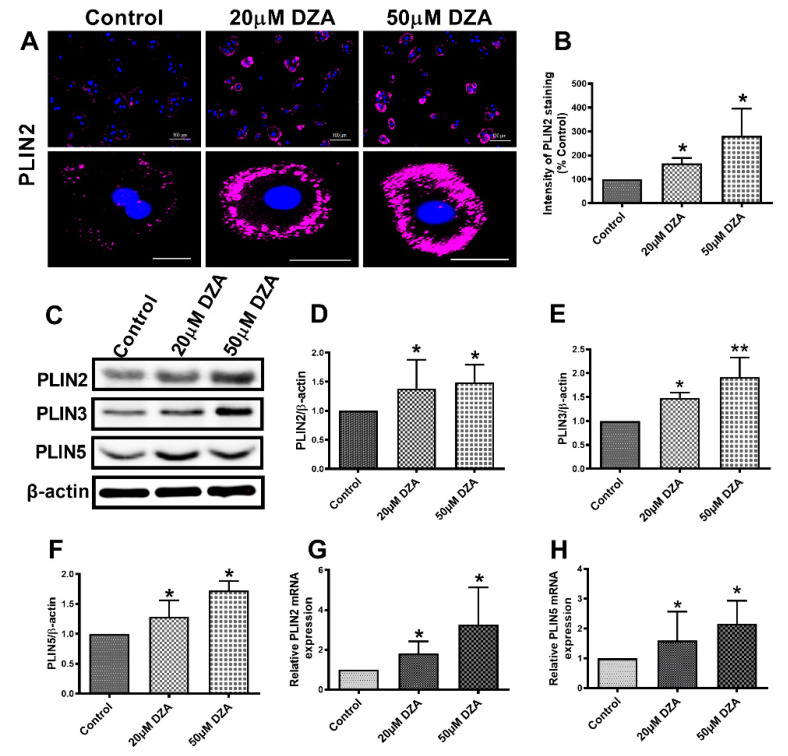
The expression of PLINs in control and DZA-treated hepatocytes. (**A**) Images of PLIN2 staining in representative control and DZA-treated hepatocytes; scale bar 100 µm (upper panel) and 20 µm (lower panel). (**B**) The fluorescence intensity of PLIN2 expression in each group of cells normalized to % of control cells, *n* = 3 experiments; images of 30 random cells from each treatment group per experiment were captured and analyzed. (**C**) Representative Western blot showing PLIN2, PLIN3 and PLIN5 levels in control and DZA-treated cells. (**D**) Immunoblot analysis summarizing the protein band density of PLIN2, (**E**) PLIN3 and (**F**) PLIN5 to β-actin, *n* = 3 experiments. (**G**) Relative PLIN2 mRNA and (**H**) PLIN5 mRNA expression in control and DZA-treated hepatocytes, *n* = 4–7 experiments. Asterisks indicate values for DZA-treated hepatocytes are statistically different from controls (* *p* ≤ 0.05 vs. Control; ** *p* < 0.01 vs. Control).

Cellular localization assessed by immunofluorescence revealed PLIN2 on the surface of most LDs. DZA exposure enhanced PLIN2 expression by 1.5 to 2.8-fold in treated hepatocytes compared with untreated control cells (Figure 2A,B). Western blot analysis also showed an increase in PLIN2, PLIN3 and PLIN5 levels after DZA treatments (Figure 2C–F). Messenger RNA quantification revealed higher PLIN2 and PLIN5 mRNA levels after DZA treatment (Figure 2G,H) which corroborated the PLIN2 and PLIN5 immunofluorescence/immunoblot results.

### 3.3. DZA Treatment Increases Sterol Regulatory Element-Binding Protein 1 (SREBP1) and Fatty Acid Synthase (FAS) Levels

**Figure 3:**
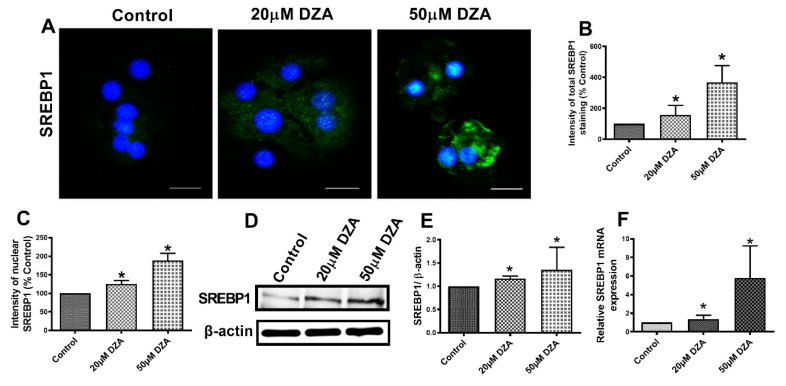
SREBP1 expression in control and DZA-treated hepatocytes. (**A**) Images of SREBP1 staining in representative control and DZA-treated hepatocytes; (scale bar 20 µm). (**B**) Fluorescence intensity of total SREBP1 expression normalized to % of control cells, *n* = 3 experiments; images of 30 random cells from each treatment group per experiment were captured and analyzed. (**C**) Fluorescence intensity of nuclear SREBP1 expression normalized to % of nuclear expression in control cells, *n* = 3 experiments; images of 30 random cells from each treatment group per experiment were captured and analyzed. (**D**) Representative Western blot showing SREBP1 levels in control and DZA-treated cells. (**E**) Immunoblot analysis of protein band density of SREBP1 relative to β-actin, *n* = 4 experiments. (**F**) Relative SREBP1 mRNA expression in control and DZA-treated hepatocytes, *n* = 6 experiments. Asterisks indicate values for DZA-treated hepatocytes are statistically different from controls (* *p* ≤ 0.05 vs. Control).

**Figure 4:**
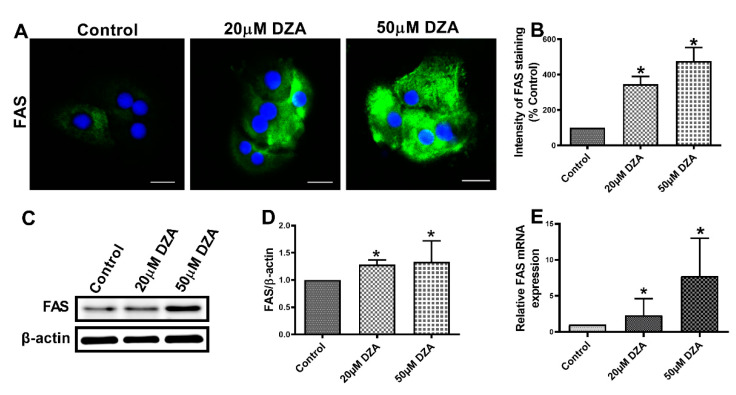
FAS expression in control and DZA-treated hepatocytes. (**A**) Images of FAS staining in representative control and DZA-treated hepatocytes; (scale bar 20 µm). (**B**) The fluorescence intensity of FAS expression normalized to % of control cells, *n* = 3 experiments; images of 30 random cells from each treatment group per experiment were captured and analyzed. (**C**) Representative Western blot showing FAS levels in control and DZA-treated cells. (**D**) Immunoblot analysis summarizing the protein band density of FAS to β-actin, *n* = 3 experiments. (**E**) Relative FAS mRNA expression in control and DZA-treated hepatocytes, *n* = 7 experiments. Asterisks indicate values for DZA-treated hepatocytes are statistically different from controls (* *p* ≤ 0.05 vs. Control).

SREBPs are a family of transcription factors known to stimulate expression of genes encoding key lipogenic enzymes [22]. We sought to determine whether DZA influences SREBP1 protein and mRNA content in control and DZA-treated hepatocytes. Immunofluorescence analyses revealed a dose-dependent rise in total SREBP1 expression (Figure 3A,B) and elevated nuclear localization (Figure 3A,C) with DZA treatments. Western blot and RT-PCR analyses revealed that DZA treatments increased SREBP1 protein (Figure 3D,E) and its mRNA (Figure 3F), compared with control cells, corroborating our immunofluorescence results. As reported, FAS is the critical enzyme responsible for de novo fatty acid synthesis [23]. Immunofluorescence, Western blot and RT-PCR analyses revealed that FAS protein (Figure 4A–D) and its mRNA levels (Figure 4E), were both significantly increased in DZA-treated hepatocytes compared with control hepatocytes.

### 3.4. DZA Treatment Increase Cell Death-Inducing DNA Fragmentation Factor Alpha-like Effector C (CIDEC) and Fatty Acid Binding Protein 4 (FABP4) Levels

**Figure 5:**
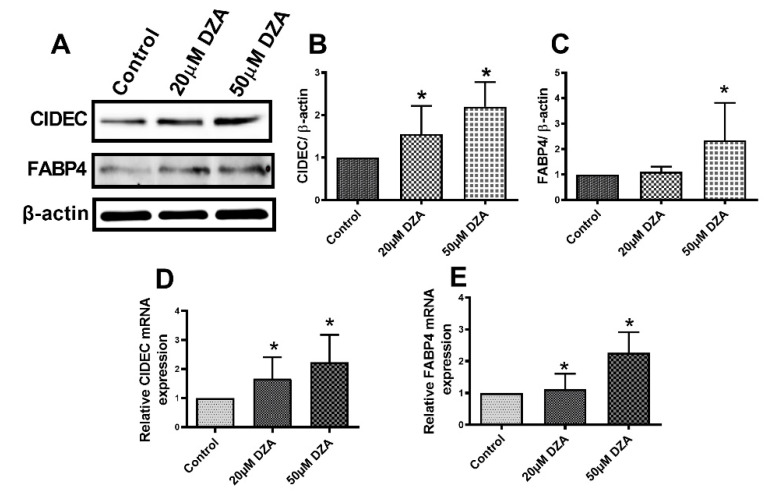
CIDEC and FABP4 expression in control and DZA-treated hepatocytes. (**A**) Representative Western blot showing CIDEC and FABP4 levels in control and DZA-treated hepatocytes. (**B**) Immunoblot analysis summarizing the protein band density of CIDEC to β-actin, *n* = 3 experiments and (**C**) FABP4 to β-actin, *n* = 3 experiments. (**D**) Relative CIDEC mRNA and (**E**) FABP4 mRNA expression in control and DZA-treated hepatocytes (*n* = 7 experiments). Asterisks indicate values for DZA-treated hepatocytes are statistically different from controls (* *p* ≤ 0.05 vs. Control).

CIDEC plays an important role in promoting LD fusion [24], while FABP4 regulates intracellular lipid metabolism by transporting fatty acids to different intracellular sites including LDs for storage as TGs [25]. RT-PCR and Western blot analyses revealed higher levels of CIDEC and FABP4 mRNAs and the proteins they encode in DZA-treated hepatocytes compared with untreated control hepatocytes (Figure 5A–E).

### 3.5. Lipases are Downregulated in Hepatocytes after DZA Treatment

**Figure 6:**
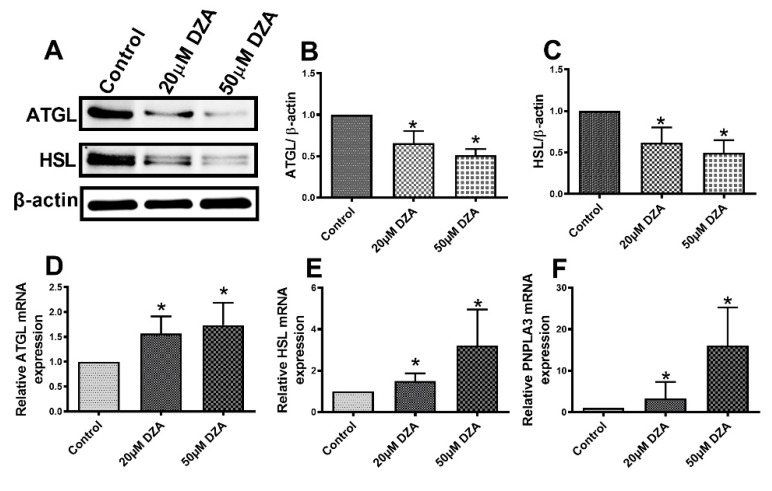
The expression of ATGL, HSL and PNPLA3 in control and DZA-treated hepatocytes. (**A**) Representative Western blot showing ATGL and HSL levels in control and DZA-treated cells. (**B**) Immunoblot analysis summarizing the protein band density of ATGL to β-actin, and (**C**) HSL to β-actin, *n* = 3 experiments. (**D**) Relative ATGL mRNA, (**E**) HSL mRNA and (**F**) PNPLA3 mRNA expression in control and DZA-treated hepatocytes (*n* = 6–7 experiments). Asterisks indicate values for DZA-treated hepatocytes are statistically different from controls (* *p* ≤ 0.05 vs. Control).

Western blot analyses revealed lower protein levels of adipose triglyceride lipase (ATGL) and hormone sensitive lipase (HSL) with DZA treatments (Figure 6A–C). Interestingly, we observed higher levels of the mRNAs encoding both these lipases in DZA-treated cells, compared with untreated controls (Figure 6D–E). The mRNA levels encoding another bonafide LD protein, patatin-like phospholipase domain-containing 3 (PNPLA3), were also higher than controls after DZA treatment Figure 6F). However, we did not quantitatively analyze PNPLA3 protein levels because we lacked a reliable antibody.

### 3.6. DZA Affects Nascent and Preformed LDs

**Figure 7:**
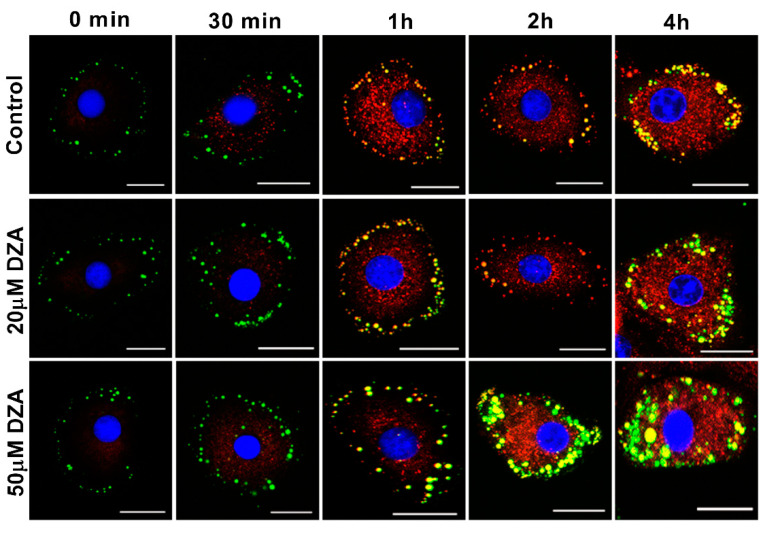
Time-bound studies depicting the effect of DZA on nascent and preformed LDs. Hepatocytes were incubated with 50 µmol/L OA media containing BODIPY FL C12 (green florescent fatty acid) overnight, followed by incubation with different concentrations (20 µm and 50 µm) of DZA treatment along with BODIPY 558/568 C12 (red fluorescent) for different time intervals. Control and DZA-treated cells were then fixed and mounted onto coverslips. Images of representative hepatocyte captured at 0 min, 30 min, 1, 2 and 4 h depicting fusion of nascent and preformed LDs in DZA-treated cells at increasing time intervals; scale bar 20 µm.

We next determined whether DZA promotes de novo LD formation and/or fusion of preformed LDs. To this end, hepatocytes in culture were incubated with green fluorescently labelled fatty acid-supplemented OA/BSA media overnight to label LDs. The cells were washed and then chased for up to 4 h in the presence or absence of DZA with a different red fluorescent-labelled fatty acid in OA/BSA medium to stain the newly formed LDs. images were captured by fluorescence microscopy at 0, 0.5, 1, 2, and 4 h of incubation, (Figure 7). Images at 30 min revealed that newly formed, red fluorescent-labelled structures were present in regions that differed in location from preformed LDs (stained green during overnight exposure to BODIPY C12) primarily seen in the periphery of the cells. With increasing dose and time of DZA exposure, some of the preformed LDs colocalized with newly formed structures and there was a simultaneous increase in the size of LDs compared with control cells. Similarly, 4 h of DZA treatment showed larger LDs that likely resulted from fusion of preformed LDs as well as fusion of the preformed LDs with nascent LDs. It is also possible that the red fluorescent fatty acid directly incorporated into preformed LDs. Collective analysis of these images suggests that DZA treatment induces molecular alterations, which promote the fusion of preformed and nascent LDs in hepatocytes.

## 4. Discussion

Our study reveals that elevated intracellular SAH levels caused by DZA exposure increase hepatocellular TGs, thereby mimicking conditions characteristic of alcohol-induced steatosis. DZA’s effects occurred largely by (1) increasing TG biosynthesis, (2) decreasing TG lipolysis, and (3) enhancing LD fusion events, thereby expanding the volume of existing LDs. We previously reported that alcohol-induced increases in liver TG and the accumulation of LDs are associated with methylation defects caused by higher levels of intracellular SAH and a lower SAM:SAH ratio [1,2,12,13,14]. We have also shown that treatment with betaine, which blocks the ethanol-induced rise in intracellular SAH and normalizes the reduced SAM:SAH ratio, prevents steatosis and LD accumulation [1,12,13,14]. We recently reported that hepatic LDs isolated from livers of ethanol-fed rats have altered phospholipid and protein compositions compared with LDs isolated from control rats [2]. Here, we demonstrated that overnight DZA exposure to hepatocytes mimics the effect of ethanol by promoting fat accumulation, mostly in the form of TGs stored in LDs. We further showed that DZA treatment, which elevates intracellular SAH [17] causing a lower methylation potential [1,2,12,13,14], enhances lipogenic and reduces lipolytic pathways by elevating key lipogenic proteins and transcription factors while reducing the levels of important lipases.

Increased hepatic accumulation and the prolonged storage of TGs within LDs is characteristic of fatty liver diseases [26], including ALD [1,2,12,13,14]. LDs are ubiquitous cellular organelles for lipid storage, which are composed of neutral lipids bounded with a phospholipid monolayer that contains proteins, the most abundant of which are the PLINs [27]. PLIN2 is one of the proteins, which stabilizes LDs and used as a surrogate marker for LD size [28]. Overexpression of PLIN2 enhances LD accumulation in hepatocytes and it also plays a major role in hepatic lipid sequestration [27]. Similarly, PLIN3 has a significant role in promoting hepatic steatosis, as elevated levels of both PLIN2 and PLIN3 are reported in both human fatty liver biopsies [29] and animal models of alcohol-related fatty liver disease [2,5,6,28,29,30,31,32]. Importantly, ablation of these PLINs causes significant increases in VLDL secretion [33] to improve hepatic steatosis [33,34,35]. PLIN5 is another LD family of protein that is highly expressed in oxidative tissues including liver [36]. Trevino et al. reported that a rise of PLIN5 in the liver drives lipid accumulation [37] by impairing lipolysis through its interaction with lipolysis regulatory proteins [38]. Here, DZA alone enhanced expression of PLIN2, PLIN3 and PLIN5 in association with increased LD accumulation in isolated, cultured hepatocytes. The rise in these PLINs by DZA treatment is similar to those reported in livers of alcohol-fed rats and mice [2,5,6,31,32].

Treatment with DZA caused a reduction in the contents of the lipolytic enzymes ATGL and HSL. These major lipases catalyze sequential steps in LD lipolysis [39,40] and their deficiencies cause LD accumulation [41,42]. Interestingly, here, we report a disconnect between the protein and mRNA levels of ATGL and HSL. We observed that, relative to controls, DZA treatment elevated ATGL and HSL mRNA levels, while those of the actual lipase proteins were lower than controls. While we do not know the reason(s) for these discordant results, the lower ATGL and HSL protein levels observed after DZA treatment are consistent with reduced lipolysis. Here, we also observed that the mRNA levels of PNPLA3, a lipase associated with lipid droplet membranes and which shows nonspecific triglyceride hydrolase activity [43], increases after DZA treatment. Genome-wide association studies support a role of PNPLA3 as a modifier of ALD [44,45]. Both acute and chronic alcohol exposure increase liver PNPLA3 expression in association with elevated hepatic TG content, indicating that PNPLA3 has a lipogenic role [46]. Elevated PNPLA3 expression in rat hepatocytes after DZA treatment mimics the observations made in alcohol-fed rats and isolated hepatocytes exposed to 100 mmol/L alcohol for 24 h in vitro [46].

SREBP1 is a transcription factor that plays an important role in lipogenesis by regulating the expression of genes involved in hepatic cholesterol, fatty acid, and TG biosynthesis [47]. Specifically, SREBP1 translocates from the cytoplasm into the nucleus, where it binds to steroid response elements in the promotor regions of a number of genes that encode enzymes involved in cholesterol and TG synthesis leading to the generation of fatty liver [48,49]. Here, the increased expression and nuclear translocation of SREBP1 in hepatocytes treated with DZA promoted lipogenesis in a manner that was similar to that of alcohol-induced SREBP1 nuclear localization [50] and activation [51].

Another LD-associated protein, CIDEC (a.k.a. fat-specific protein 27), was identified prior to the other family members, based upon its induction during adipogenesis [52]. CIDEC plays an important role in promoting LD fusion [24], generating supersized LDs that presumably act as a lipolytic barrier by hindering the access of lipases to the LD TG stores [53,54]. Increased hepatic CIDEC expression is observed in fatty livers of mice [55,56], alcohol-fed rats (unpublished observations) and obese patients [57], and hepatocyte-specific loss of CIDEC ameliorates hepatic steatosis [56]. DZA treatment indeed caused the accumulation of larger-sized LDs, as judged by BODIPY 493/505 staining and by the time-bound (i.e., “pulse-chase”) study that followed the fusion of preformed LDs with differentially labelled nascent LDs. The accumulation of larger-sized LDs by DZA treatment observed here likely resulted from higher CIDEC mRNA and protein levels, which facilitated LD fusion within the backdrop of the increased lipogenesis and decreased lipolysis of LD TG stores.

FABP4 regulates intracellular lipid metabolism by transporting fatty acids to the nucleus for transcriptional regulation, to mitochondria for β-oxidation, and to lipid droplets for storage as TGs [25]. FABP4 is an adipocyte-specific fatty acid binding protein whose expression increases in hepatocytes after alcohol consumption [58]. Here, DZA treatment increased both the FABP4 mRNA and protein expressions. Such induction likely contributed to increased uptake and trafficking of fatty acids for esterification and storage in LDs.

**Figure 8:**
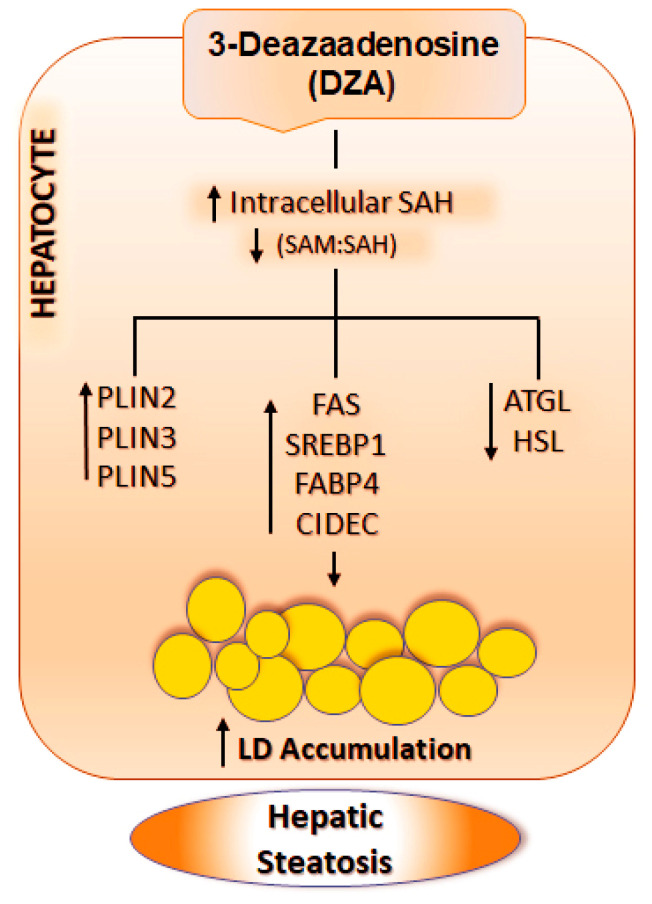
Schematic representation of the mechanism by which DZA results in LD accumulation in hepatocytes. DZA treatment causes a rise in intracellular SAH levels and reduces the SAM:SAH ratio in hepatocytes, which in turn, increases PLINs, enhances lipogenesis and decreases lipolysis. Collectively, these events promote increased LD accumulation in hepatocytes, characteristic of hepatic steatosis. The abbreviations used are as follows: SAH, S-adenosyl homocysteine; SAM, S-adenosylmethionine; PLINs, Perilipin; FAS, Fatty acid synthase; SREBP1, Sterol regulatory element-binding protein 1; FABP4, Fatty Acid-Binding Protein 4; CIDEC, Cell death inducing DFFA like effector c; ATGL, Adipose triglyceride lipase; HSL, Hormone-sensitive lipase.

In summary, here we report that DZA mimicked both acute and chronic alcohol consumption [1,2,12,13,14,46,59,60] by causing steatosis in hepatocytes. Our data strongly indicate that DZA treatment modulates molecular events responsible for the growth and accumulation of LDs in hepatocytes. Specifically, we show that DZA treatment increased intracellular SAH which, in turn, (i) upregulates lipogenesis; (ii) increases uptake and mobilization of fatty acids by increasing the expression of fatty acid binding protein; and (iii) reduces triglyceride turnover by lowering lipase levels and raising the expression of anti-lipolytic factors such as PLINs and CIDEC. Collectively, these events (schematically shown in Figure 8) promote LD accumulation characteristic of hepatic steatosis. We recognize that a limitation of this study is that all experiments were conducted on primary hepatocytes, which does not accurately recapitulate the heterotypic cell–cell complex interactions that occur during ALD pathogenesis. However, hepatocytes are the principal sites of LD accumulation during development of hepatic steatosis as well as the liver cell type most affected by a rise in intracellular SAH. To summarize, our study shows that the elevation of intracellular SAH levels is sufficient to alter methylation reactions and promote fat accumulation in hepatocytes similar to that seen after alcohol administration [1,2,12,13,14]. We conclude that ethanol-induced alteration in the methionine metabolic pathway, especially an increase in hepatocellular SAH, plays a crucial role in promoting LD accumulation, a characteristic feature of alcoholic steatosis.

## Abbreviations

Adipose triglyceride lipase (ATGL); Alcohol-related liver disease (ALD); Boron-dipyrromethene (BODIPY); Bovine serum albumin (BSA); Cell death-inducing DNA fragmentation factor alpha-like effector (CIDEC); C4′,6-diamidino-2-phenylindole (DAPI); 3-deazaadenosine (DZA); Fatty acid binding protein 4 (FABP4); Fatty acid synthase (FAS), Hormone sensitive lipase (HSL); Lipid droplets (LDs); Messenger RNA (mRNA); Patatin-like phospholipase domain-containing 3 (PNPLA3); Perilipins (PLINs); Phosphatidylcholine (PC); Phosphatidylethanolamine (PE); Reverse transcription polymerase chain reaction (RT-PCR); S-adenosylmethionine (SAM); S-adenosylhomocysteine (SAH); Sterol regulatory element-binding protein 1 (SREBP1), Triglyceride (TG).
